# Protocol for a pragmatic stepped wedge cluster randomized clinical trial testing behavioral economic implementation strategies to increase supplemental breast MRI screening among patients with extremely dense breasts

**DOI:** 10.1186/s13012-023-01323-x

**Published:** 2023-11-24

**Authors:** Anne Marie McCarthy, Claudia Fernandez Perez, Rinad S. Beidas, Justin E. Bekelman, Daniel Blumenthal, Elizabeth Mack, Anna-Marika Bauer, Sarah Ehsan, Emily F. Conant, Bernadette C. Wheeler, Carmen E. Guerra, Linda W. Nunes, Peter Gabriel, Abigail Doucette, E. Paul Wileyto, Alison M. Buttenheim, David A. Asch, Katharine A. Rendle, Rachel C. Shelton, Oluwadamilola M. Fayanju, Sue Ware, Martina Plag, Steven Hyland, Tracy Gionta, Lawrence N. Shulman, Robert Schnoll

**Affiliations:** 1 Perelman School of Medicine, University of Pennsylvania, Philadelphia, PA USA; 2 Penn Center for Cancer Care Innovation, Abramson Cancer Center, Penn Medicine, Philadelphia, PA USA; 3 Present Address: Department of Biostatistics, Epidemiology, and Informatics, University of Pennsylvania, Blockley Hall, 423 Guardian Drive, Philadelphia, PA 19104 USA; 4 Feinberg School of Medicine, Northwestern University, Chicago, IL USA; 5 Center for Interdisciplinary Research On Nicotine Addiction, University of Pennsylvania, Philadelphia, PA USA; 6 School of Nursing, University of Pennsylvania, Philadelphia, PA USA; 7 Mailman School of Public Health, Columbia University, New York, NY USA

**Keywords:** Supplemental breast MRI, Breast density, Implementation science, Behavioral economics, Nudges, Stepped wedge design, Pragmatic trial

## Abstract

**Background:**

Increased breast density augments breast cancer risk and reduces mammography sensitivity. Supplemental breast MRI screening can significantly increase cancer detection among women with dense breasts. However, few women undergo this exam, and screening is consistently lower among racially minoritized populations. Implementation strategies informed by behavioral economics (“nudges”) can promote evidence-based practices by improving clinician decision-making under conditions of uncertainty. Nudges directed toward clinicians and patients may facilitate the implementation of supplemental breast MRI.

**Methods:**

Approximately 1600 patients identified as having extremely dense breasts after non-actionable mammograms, along with about 1100 clinicians involved with their care at 32 primary care or OB/GYN clinics across a racially diverse academically based health system, will be enrolled. A 2 × 2 randomized pragmatic trial will test nudges to patients, clinicians, both, or neither to promote supplemental breast MRI screening. Before implementation, rapid cycle approaches informed by clinician and patient experiences and behavioral economics and health equity frameworks guided nudge design. Clinicians will be clustered into clinic groups based on existing administrative departments and care patterns, and these clinic groups will be randomized to have the nudge activated at different times per a stepped wedge design. Clinicians will receive nudges integrated into the routine mammographic report or sent through electronic health record (EHR) in-basket messaging once their clinic group (i.e., wedge) is randomized to receive the intervention. Independently, patients will be randomized to receive text message nudges or not. The primary outcome will be defined as ordering or scheduling supplemental breast MRI. Secondary outcomes include MRI completion, cancer detection rates, and false-positive rates. Patient sociodemographic information and clinic-level variables will be examined as moderators of nudge effectiveness. Qualitative interviews conducted at the trial’s conclusion will examine barriers and facilitators to implementation.

**Discussion:**

This study will add to the growing literature on the effectiveness of behavioral economics-informed implementation strategies to promote evidence-based interventions. The design will facilitate testing the relative effects of nudges to patients and clinicians and the effects of moderators of nudge effectiveness, including key indicators of health disparities. The results may inform the introduction of low-cost, scalable implementation strategies to promote early breast cancer detection.

**Trial registration:**

ClinicalTrials.gov NCT05787249. Registered on March 28, 2023.

**Supplementary Information:**

The online version contains supplementary material available at 10.1186/s13012-023-01323-x.

Contributions to the literature

- This study will evaluate the effects of nudges (informed by behavioral economics) directed to patients and/or clinicians on the uptake of supplemental breast MRI screening among patients with extremely dense breasts.
- Insights gained from this study may be able to inform future dissemination of low-cost and scalable approaches for boosting engagement with supplemental breast MRI screening.
- This study uses rapid-cycle approaches to support the implementation of a clinical trial assessing engagement with supplemental breast MRI screening and will use mixed-methods approaches informed by existing health equity frameworks to evaluate factors that facilitate or impede reach.

## Background

A key risk factor for breast cancer is breast density, a measure of fibroglandular tissue compared to fat on mammographic imaging [1]. The risk of developing breast cancer is up to five times higher among women with extremely dense breasts than among women with entirely fatty breasts [1–4]. Furthermore, dense breast tissue can reduce mammography sensitivity by masking tumors [5–7]. As such, there is growing recognition that for some women, mammography may not be sufficient for breast cancer surveillance. Indeed, the European Society for Breast Imaging recommends supplemental screening via breast magnetic resonance imaging (MRI) for women with dense breasts [8], and the American College of Radiology recommends breast MRI for women with dense breasts who desire supplemental screening [9].

Approximately 7.4% of American women aged 40 to 74 years (equating to about five million people) have extremely dense breasts [10], with variation based on race/ethnicity, age, and BMI [11, 12]. Randomized controlled trials demonstrate that supplemental breast MRI can reduce the risk of interval cancers (i.e., those diagnosed after a non-actionable mammogram and before the next screening exam) compared to mammography alone for women with extremely dense breasts [13–16]. Simulation models suggest that adding MRI to every other mammography round for these women would save 7 additional lives per 1000 women [17] and that MRI can be cost-effective [17–19]. Adding MRI to routine screening may increase financial burdens, but recent advances in abbreviated MRIs [20] and governmental support [21, 22] suggest decreasing future costs. Increased sensitivity of breast MRI improves detection, but it can result in false positives which may lead to unnecessary biopsies and downstream costs.

A recent meta-analysis concluded that, due to its increased efficacy in detecting incremental and invasive cancers, breast MRI was the best supplemental screening method for women of average or intermediate risk with dense breasts [23]. Despite potential benefits, uptake of supplemental breast MRI remains low. Only about 10% of women with dense breasts are screened with MRI [24–27], representing a clear missed opportunity to identify cancers earlier.

Significant health inequities exist in supplemental MRI uptake as well, with Black and Hispanic women receiving supplemental screening less than non-Hispanic white women [28, 29]. This race-based gap is significant since Black women are more likely to have triple-negative breast cancer [30, 31], a more aggressive breast cancer subtype which is harder to detect via mammography [32–34]. Racial inequities in MRI uptake may relate to differential access. Specialist clinicians order supplemental screening more than generalists, but they are less likely to see Black and Hispanic patients [29, 35]. Additionally, Black patients must travel significantly farther to get MRI screening [36]. Finally, differences in health insurance coverage can widen the gaps in supplemental screening rates [37]. Overall, efforts to expand supplemental breast MRI screening must consider issues faced by minoritized populations to promote health equity.

Barriers to increasing the reach of supplemental breast MRI are multi-level. Patient-level barriers include financial burden, anxiety about undergoing MRI procedures and receiving results, and lack of awareness [36, 38–42]. Most states require that patients be notified of their breast density [43] and a U.S. Food & Drug Administration mandate going into effect in 2024 will scale this nationwide [44]. New laws in Pennsylvania, the location of this study, also mandate insurance coverage of supplemental screening for women with dense breasts [21, 22]. However, while necessary, policy-level changes are often insufficient. They show mixed effects for boosting awareness, are tied to only modest increases in screening [24, 25, 45–47], and may cause confusion and undue anxiety because breast density notifications are often too technical and complex for patients [48–54]. Clinicians tend to express discomfort in discussing breast density and supplemental screening with patients [35, 55, 56]. This may be due to the absence of consistent guidelines, worries about financial costs or potential false positives, or concerns about MRI screening in this context [45, 57–59]. They may also be unaware of the clinical importance of breast density or the existence of new legislation mandating insurance coverage [55].

Together, these barriers can lead to uncertainty about the value of supplemental breast MRI. Uncertainty can cause decision-makers to rely on heuristics [60] that reduce evidence-based care and exacerbate health inequities [61]. Availability bias (an over-reliance on information that is salient in one’s mind) [60] and omission bias (a preference for letting harm happen due to inaction rather than being responsible for harm resulting from taking action) [62] are relevant here. If patients are not aware of the importance of breast density in predicting breast cancer, they may be less interested in pursuing breast MRI. Conversely, making breast MRI more salient and delivering information at the right time could inspire interest [63]. Similarly, clinicians may believe that doing nothing is preferable to potentially causing harm by making patients deal with the costs of a breast MRI or the consequences of a false positive. However, focusing their attention on the value of early action, new insurance coverage mandates, and recent data on efficacy could mitigate this concern. Nudges, or changes to the presentation of choices to guide decision-making, can frame information accordingly to promote evidence-based care while still maintaining decision-makers’ agency [64–69]. In many cases, nudges can be automated through the electronic health record (EHR), patient portal, or text messages [70–72]. This flexibility can enable nudges to be cost-effective and be scaled up quickly [71].

This study is designed to evaluate the relative effects of patient- and clinician-directed implementation strategies informed by behavioral economics on the ordering and scheduling of supplemental breast MRI. Patients with extremely dense breasts who may benefit from breast MRI screening will be identified, and nudges will be delivered to these patients and/or their clinicians. Given inequities in supplemental screening, sociodemographic variables (e.g., race and ethnicity) and organizational characteristics (e.g., clinician specialty, distribution of insurance status) ascertained from the EHR will be assessed as moderators of the nudges’ impact. Finally, a qualitative component will identify barriers and facilitators of the implementation strategies, including factors that may mitigate or exacerbate health inequities.

## Methods

### Study setting and population

The study setting for this project will be mammography sites within our Implementation Laboratory (iLab) [73]: Penn Center for Advanced Medicine (part of the Hospital of the University of Pennsylvania), Penn Presbyterian Medical Center, Pennsylvania Hospital, and Penn Medicine Radnor (an ambulatory site of care). In support of these four sites, we identified 32 primary care and OB/GYN practices with approximately 1100 clinicians who may order mammograms and may be eligible to receive the clinician nudge promoting breast MRI ordering. In spite of recent legislation mandating insurance coverage of breast MRI for patients with extremely dense breasts [21], an initial gap analysis of our health system showed that MRI uptake among women with extremely dense breasts was around 8%. Additionally, the rate was about three times lower among Black women than among White women. Based on the data on mammogram completion from January to December 2022, the target enrollment for this study will be approximately 1600 patients with extremely dense breasts.

To be eligible, patients must be 40–74 years old, have had a non-actionable mammogram in the past 6 months, have had that mammogram at one of the study sites, have been identified as having extremely dense breasts on that mammogram, and have a valid mobile phone number to receive the patient nudge. They also must not have had a prior history of breast cancer or have had a breast MRI within the last 2 years. For clinicians to be eligible, they must have ordered the initial screening mammogram, be employed by a clinic in one of the iLab practices, and have access to our health system’s EHR. Clinicians will be clustered based on the clinics in which they practice. Clinics will be grouped into clusters based on administrative departments and care patterns to minimize potential contamination resulting from clinicians providing care at more than one clinic. Clinic groups will be used as the unit of randomization for the stepped wedge design, and the order in which steps begin to receive the nudge will be randomized.

### Study design and duration

**Fig. 1:**
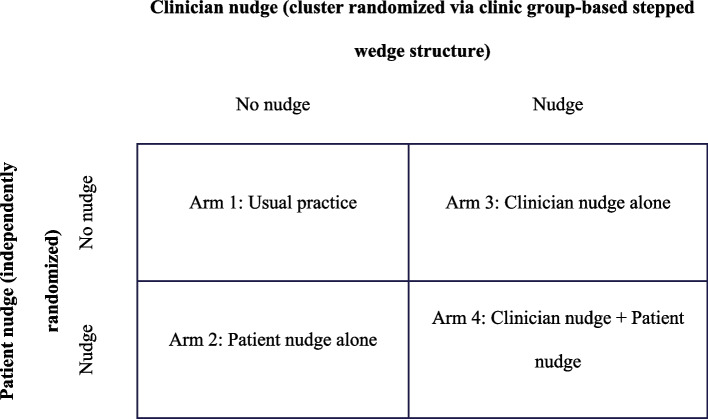
Study design

**Fig. 2:**
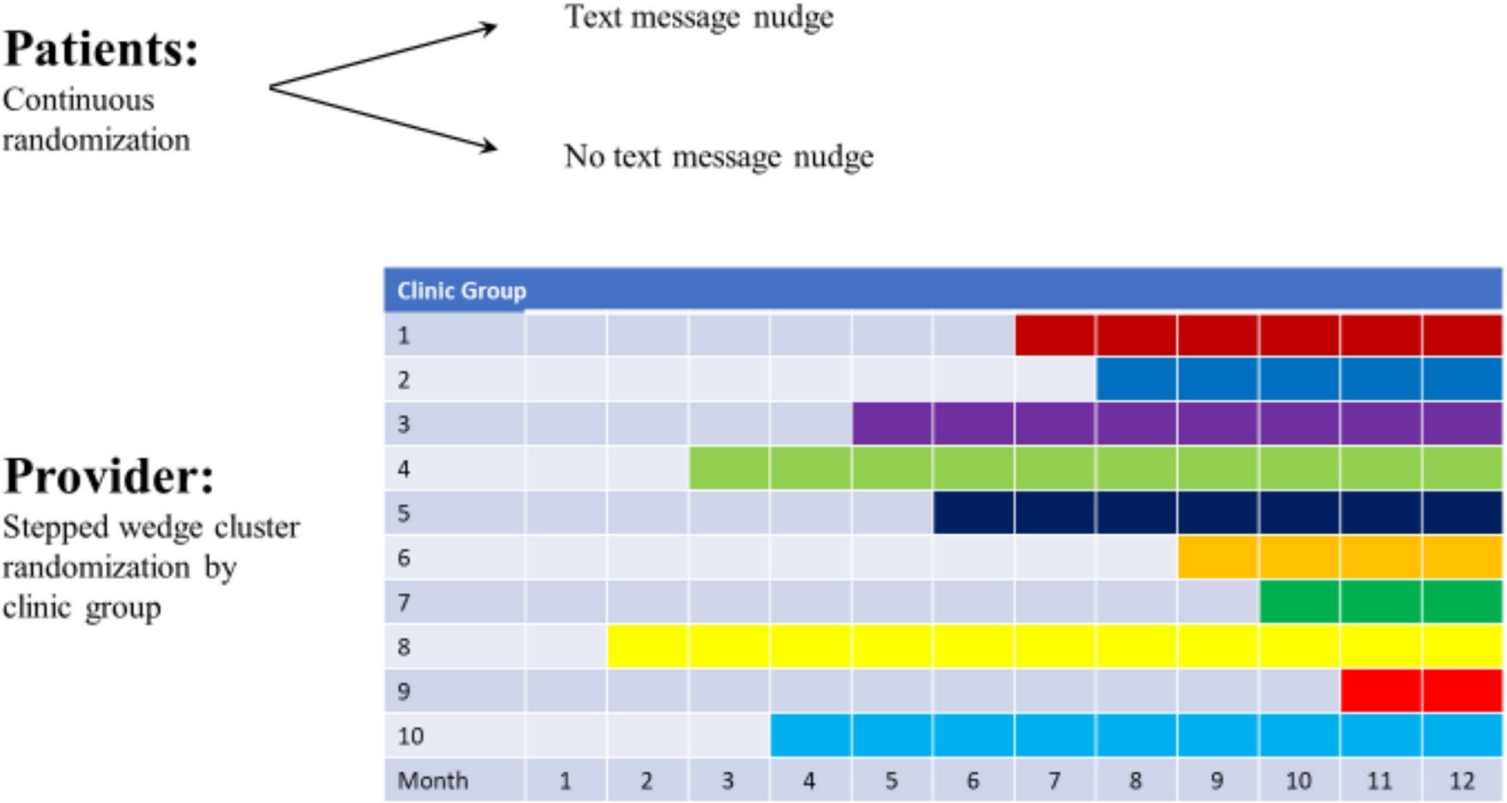
Randomization scheme

**Fig. 3:**
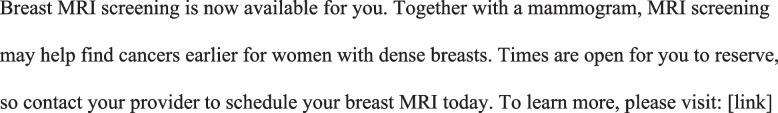
Patient nudge

A pragmatic stepped wedge cluster randomized clinical trial with embedded patient-level randomization will be conducted to test the effects of behavioral economics-informed nudges to clinicians, patients, both, or neither on supplemental breast MRI ordering and scheduling for patients with extremely dense breasts. Figure 1 outlines the 2 × 2 factorial study design, resulting in a usual care arm that will not receive either nudge, a patient nudge only arm, a clinician nudge only arm, and a “both nudges” arm. Figure 2 displays the independent randomization approach for patients and clinicians. Eligible patients will be identified continuously throughout the study after a non-actionable mammogram showing extremely dense breasts. These patients can be independently and individually randomized to get the patient nudge as soon as the study starts. Patient nudges will be sent via secure text messaging and will contain a timely and relevant message encouraging patients to speak with their clinicians about breast MRI (see Fig. 3).

Primary care and OB/GYN practices within our academic health system will be cluster randomized by clinic group to 10 different steps. The steps determine the order in which clusters make the transition from standard care to clinician nudges. Clinic groups were developed based on clinics with shared administrative departments, similar care patterns, and overlapping clinician populations. Clusters based on clinic group will reduce potential contamination and facilitate timely dissemination of nudges and educational materials. Based on retrospective data, we expect about 90% of clinicians to only see patients in one clinic group.

**Fig. 4:**
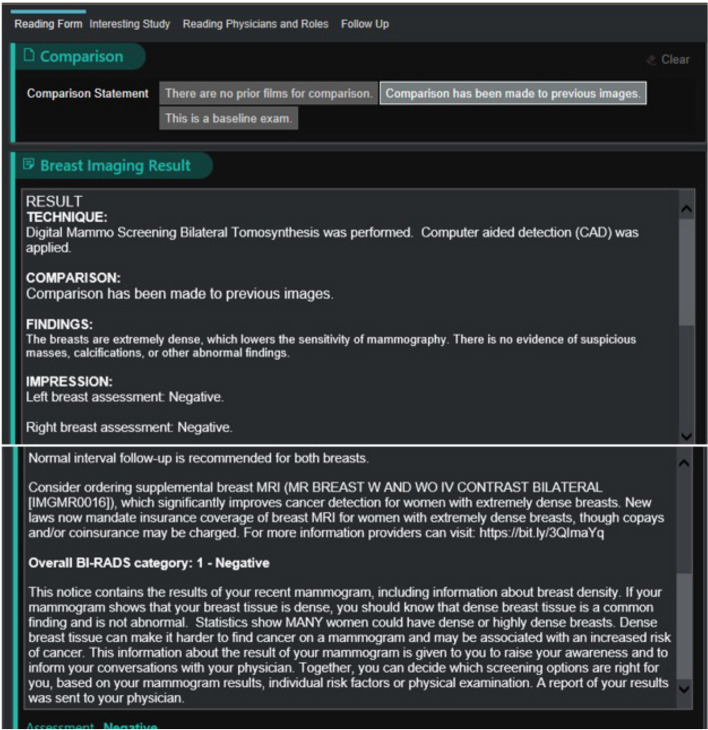
Mammogram report featuring the clinician nudge in the impression section

At all mammography sites except one, the clinician nudge will consist of text embedded within the “impression” section (where mammogram results are displayed) of the routine report clinicians receive after a screening mammogram. As detailed in Fig. 4, the note will address barriers to MRI ordering (e.g., cost) and feature a timely call to action for clinicians to order supplemental breast MRI for patients with extremely dense breasts. Due to technical restrictions related to radiology report generation and differing software across sites, clinicians of patients who receive their mammogram at one mammography site will receive the same message but via an EHR-based in-basket system. After the trial, discussions with participants will be completed and qualitatively coded to identify barriers and facilitators of the implementation strategies.

**Fig. 5:**
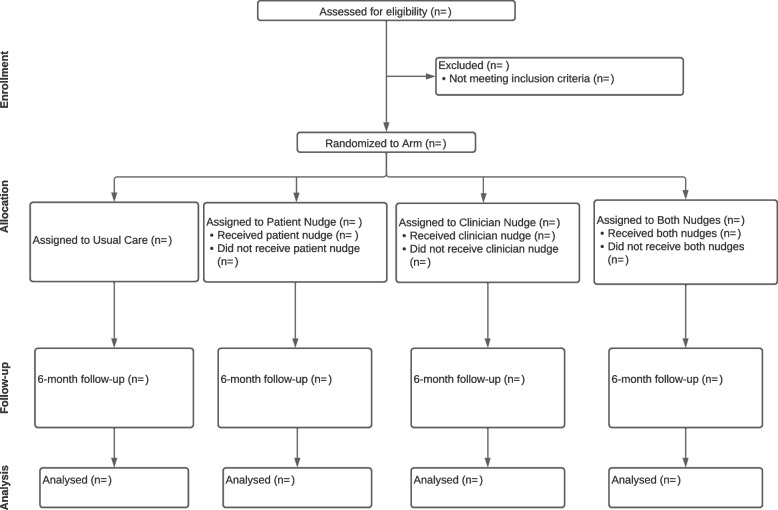
CONSORT flow diagram

The study duration will be approximately 18 months. Nudges will be deployed over the course of a year, with patients receiving nudges within 1 month of their initial mammogram. In the first month, no steps will be randomized to receive the clinician nudge. With each subsequent month, one step will be added to receive it, and by the final month, all clinic groups will be receiving the clinician nudge (see Fig. 2). After this 12-month period, 6 months will be allotted for outcomes (e.g., scheduling and completion of MRI appointments) to be ascertained and analyzed (Fig. 5). The primary outcome variable will be whether a clinician orders a supplemental breast MRI for their patient with extremely dense breasts or whether a patient schedules a supplemental breast MRI within 6 months of the nudge. Based on prior studies [74], our primary hypothesis is that the “both nudges” arm will yield significantly higher ordering and scheduling of supplemental MRI than the usual care arm. Clinician nudges may promote more clinician ordering, as found in a prior trial on tobacco use treatment referral in our center [75], while patient nudges may promote more patient scheduling.

### Overview of study procedures

The first step for boosting the uptake of supplemental breast MRI screening is to identify the patients who may benefit from it. The study sample was limited to patients with only extremely dense breasts due to the highest risk of interval cancer and the strongest data from the literature about MRI’s benefits for those with extremely dense breasts [4, 14]. Breast density is available as a hard-coded (rather than user-generated) field through the Epic Radiant Radiology Information System (Epic Systems Corporation, Verona, WI). Using this information and the above inclusion criteria, an EHR phenotyping algorithm was developed to identify patients eligible for this trial. Patients identified by this phenotyping algorithm will be linked with our informatics team, who will handle nudge dissemination. Nudges will be deployed to patients and clinicians based on randomization to trial arm. If randomized to an arm with a clinician nudge (clinician nudge or “both nudges” arms), clinicians will be sent the nudge embedded within the mammogram report or through an EHR-based in-basket message. If randomized to an arm with a patient nudge (patient nudge or “both nudges” arms), patients will have the potential to be sent the nudge via secure text message through Penn Way to Health, an evidence-based patient engagement platform [76, 77]. Patients will receive initial messages asking them to confirm their identity and seeing if they want to learn more about cancer screening. If they confirm their identity and express interest, they will receive the nudge. If patients do not respond to the initial message, reminders will be sent 3 and 7 days later. No further reminders will be sent, but non-responders will still be included in the intent-to-treat analysis. The first text message will be sent at least 2 days after the mammogram report is shared with the patient to reduce the chances that it is the first time the patient is contacted after their mammogram but no more than 2 weeks after the mammogram to ensure that the message remains timely.

### Formative work using rapid cycle approaches (RCA)

We used RCAs to optimize and de-risk the content and method of delivery of the patient and clinician nudges. RCAs enable innovators to learn via preliminary tests and quickly iterate on their interventions [78–81]. They provided a cost-effective way to enhance the nudges and ensure their feasibility prior to our trial. Table 1 provides more detail on the processes implemented as part of the RCAs. Meetings with key patient and clinician partners and subject matter experts in behavioral economics, implementation science, and health equity helped guide decisions about nudge delivery and content. This ensured that the nudges addressed key barriers (e.g., cost) were at appropriate readability levels and were relevant for patients with varied racial/ethnic backgrounds. In addition, pilot tests were conducted with 180 patients to compare three nudges reflecting different behavioral barriers to scheduling a supplemental breast MRI. The final nudges used in the trial were selected based on these RCAs.

**Table 1:** Rapid cycle approaches (RCA) to develop, de-risk, and optimize nudges

Domain	Initial approach	Iterative work	Output
Clinician nudge	Best practice alert (BPA) encouraging clinicians to order breast MRI screening Key questions: • When is the best time to send nudges to clinicians? • What are the best strategies for sending a nudge to clinicians?	Approach: Sessions with experts in behavioral economics, implementation science, breast cancer screening, and health equity; technical feasibility tests with informatics and radiology leads Key feedback: • BPAs can interfere with clinical workflows, may be perceived as annoying, and can contribute to alert fatigue for clinicians • Integrating a prompt-to-order screening into the mammogram report would be ideal for clinical workflows • When reviewing mammogram reports, the “impression” section is often the first place clinicians look • Embedding text into reports and subsequent randomization of this language is not feasible at certain sites • Clinicians could benefit from additional educational materials	• Rather than a BPA, clinician nudges were included within the “impression” section of mammogram reports to integrate this content into the clinical workflow • The standard clinician nudge was implemented at all sites but one; an EHR-based in-basket message was used at one site • A one-pager and educational website with more information about supplemental breast MRI screening was created, and study leads presented to clinical teams
Patient nudge	Nudges delivered via text message Key questions: • What are the behavioral barriers affecting the uptake of supplemental breast MRI? • What specific message content would be most salient to patients?	Approach: Meetings with experts in behavioral economics, implementation science, breast cancer screening, and health equity; review by patient advisory committee; rapid mini-pilots with patients Key feedback: • Barriers for patients include cost concerns and an overall lack of awareness about breast MRI, so nudges should highlight these issues • Additional educational material should be provided to help patients learn more about breast density and supplemental MRI • Despite the passage of state laws covering breast MRI for certain patients, insurance coverage is highly variable. There still can be costs associated with breast MRI that can make it infeasible for some patients	• Mini-pilots were conducted via text message to assess engagement rates for three potential patient-directed nudges • Key wording changes were made to increase readability and reduce confusion about costs • A new questions and answers webpage was created to provide patients with additional information • The final patient-directed nudge focused on making the relevant information salient and timely and invoked a sense of urgency and potential scarcity in a clear cue to action

### Implementation strategies

#### Patient nudge

Nudges to patients (Fig. 3) will be delivered via text message within 2 weeks of a non-actionable mammogram in which a patient is identified as having extremely dense breasts. The nudge will highlight the added value of supplemental breast MRI screening for improving early breast cancer detection, contain a call to action to encourage patients to contact their clinicians to schedule supplemental screening, and provide a link for them to learn more about breast density. Based on the results of the RCA, message content was framed to be timely and salient (by sharing relevant and potentially new information about breast MRI’s benefits) and to emphasize the value of taking action early. Patients will be able to opt out of future study-related outreach should they not want to participate. Additional file 1 includes the educational questions and answers page that is linked within the message.

#### Clinician nudge

Nudges to clinicians will be implemented as a note embedded within mammogram result reports or as an EHR-based in-basket message containing similar content. All analyses will be stratified by mammography site to account for this difference. Figure 4 shows the clinician nudge content, which is designed to serve as a timely notice to focus clinicians’ attention on the advantages and newly reduced costs of supplemental breast MRI. Clinician education and the benefits and risks of breast MRI will be shared via presentations at existing clinician meetings, informational videos, one-page information sheets (Additional file 2), and online resources.

#### Both nudges

In this arm, both the patient and clinician nudges will be implemented.

#### Usual care

In this arm, neither nudge will be deployed.

### Measures

The primary outcome will be defined as ordering or scheduling breast MRI within 6 months of the delivery of the nudge, or in the case of the usual care arm, from the time of the mammogram. Secondary measures will include whether a patient completes supplemental breast MRI screening, as well as clinical measures ascertained from the EHR for those who complete breast MRI screening, such as cancer detection rate and false-positive rate. Response rates for text messages sent to patients will be assessed as process measures.

The EHR, databases maintained by our health system, and publicly available U.S. Census data will be used to collect information on practices, clinicians, and patients that could serve as potential moderators of nudge effects or influences on potential inequities. Clinic-level details will include location and health insurance distribution. Demographics and faculty track may be collected as clinician-level data. Patient-level data will include age, race and ethnicity, health insurance status, and address. Analyses of supplemental breast MRI uptake across study arms will be stratified based on these factors as necessary, and given existing racial inequities, breast MRI uptake will be compared between Black and non-Hispanic white patients.

### Qualitative aim

After patient enrollment ends, semi-structured interviews will be conducted to understand patient and clinician experiences with the trial. These interviews will assess the program’s impact and implementation based on the domains of the updated Consolidated Framework for Implementation Research (CFIR) [82, 83]. Questions for patients will focus on implementation measures, such as the appropriateness of the nudges and supplemental breast MRI screening. In support of our center’s core health equity theme [84, 85], questions on system-level barriers impeding supplemental breast MRI uptake, such as limited health care access, financial barriers, and experiences of racism and discrimination, will be included in interviews. These discussions will be informed by existing equity-focused implementation science frameworks [86, 87]. Clinician interviews will focus on identifying facilitators and barriers to conducting supplemental breast MRI screening. Interview participants will be purposively sampled to over-represent those who may be experiencing health inequities.

### Sample size, power, and statistical analysis

Based on the initial assessments via electronic phenotyping, the target population will consist of about 1600 patients in our health system with extremely dense breasts who may benefit from supplemental breast MRI screening. Ten clusters with one to seven administrative departments each were created based on the existing care patterns. This was done to minimize contamination and facilitate the timely dissemination of nudges and educational material. We calculated power requirements by simulation using Stata 17, assuming a logistic regression model fitted using generalized estimating equations (GEE), and found our sample gives us 80% power to detect a 6% improvement in our primary outcome (e.g., from the current 8 to 14%) for patient nudges, using a two-sided type 1 error rate of 5%, for planned comparisons between each sequence. The analysis provides similar power to detect a 7% improvement from clinician nudges and a 10% difference representing the interaction between patient and clinician nudges vs. usual care.

We will analyze the changes in the rates of ordering, scheduling, and screening completion across the four arms (all binary outcomes) using logistic regression with GEE. The models will contain binary predictor terms for each arm, adjusting for time in months, and fixed effects for mammogram site. We will control for type 1 error inflation by hierarchical testing, starting with the overall model significance, followed by the effect of each nudge. Once we have fitted the main effects model, we will test for each sequence and retain terms if significant (alpha = 5%). Variability in outcomes by sequence (wedge) and moderators (particularly variables like race which may reflect health inequities) will be assessed using interaction terms within logistic regression models. We will fit an adjusted logistic regression model using the same approach described in the primary analysis. Covariates of interest available through the EHR will be added to the model, including patient-level (e.g., race), clinician-level (e.g., faculty track), and practice-level (e.g., community vs. hospital-based) data. We do have some concerns that a small number (< 10%) of clinicians see patients at more than one location. This contamination would bias the effect estimates toward the null. The primary analysis will use clinician treatment as randomized by primary location. We will address potential contamination by conducting sensitivity analyses. These will include 1 “as-treated,” basing treatment effect on the cumulative number of nudges a clinician has received, regardless of location, and 2 estimating an uncontaminated treatment effect, excluding patients seen by clinicians who ordered mammograms for patients in more than one step. Lastly, interview data will be analyzed using NVivo to identify themes regarding barriers and facilitators of nudge impact and implementation.

This study was approved by the University of Pennsylvania Institutional Review Board and registered with ClinicalTrials.gov. This is a pragmatic trial that allows clinicians to choose whether or not to order supplemental screening depending on what may be clinically appropriate for their specific patient. As such, the study presents minimal risks to patients and clinicians, so a waiver of informed consent was approved. Qualitative interviews conducted after enrollment will solicit informed consent from potential participants prior to data collection.

## Discussion

This trial will test the effects of nudges to patients, clinicians, or both, compared to usual care, as implementation strategies to increase utilization of supplemental breast MRI screening among patients with extremely dense breasts. By implementing nudges directed at both patients and clinicians, the study provides the opportunity to not only determine the effects of nudges overall, but also to evaluate the relative effects of nudges in each arm. It also builds upon our center’s prior studies [75, 88] by extending into new clinical service lines and assessing whether nudges can be effective implementation strategies in cancer screening, in addition to cancer control and treatment. This study seeks to increase the ordering and scheduling of supplemental breast MRI screening, aligning with the implementation outcome of penetration, but also to advance the integration of implementation science and behavioral economics more broadly by exploring the patient- and clinician-level factors that affect receptiveness to nudges from health systems. It will also provide more information about how patient- and clinician-directed nudges may impact health disparities in cancer screening delivery.

While the inclusion of multiple sites with diverse locations and patient populations can increase external validity, this is only a single-health system study. The results may not be generalizable to other health systems, especially those without EHR integration or with limited access to the equipment needed for breast MRI. In addition, patients may find it unexpected to receive a text message from the health system regarding their breast density and cancer screening opportunities. This could cause unnecessary anxiety for patients. To reduce the odds of the notification causing adverse emotional reactions while still maintaining timeliness, messages will be sent soon, but not immediately after, a non-actionable mammogram result. The decision to engage with supplemental breast MRI relies on input from both patients and their clinicians, so even if nudges encourage one party to pursue supplemental screening, a lack of interest from the other could mean that the MRI is not scheduled. Risks of MRI (e.g., false positives) are a crucial consideration, and our nudges allow clinicians and patients to decide the appropriate next steps after a mammogram without inhibiting their agency. Our nudges are not designed to overcome potential structural barriers such as limited capacity. If successful, this study could inform a larger trial to implement impactful strategies across multiple health systems to increase supplemental breast MRI utilization at scale. Additionally, should patients, clinicians, and health system leaders approve, the strategies could be expanded to patients with heterogeneously dense breasts.

### Supplementary Information

**Additional file 1. **Patient Education Materials.

**Additional file 2.** Clinician Education Material.

## Data Availability

Not applicable.

## Abbreviations

BMI: Body mass index
BPA: Best practice alert
CFIR: Consolidated Framework for Implementation Research
EHR: Electronic health record
GEE: Generalized estimating equations
iLab: Implementation Laboratory
MRI: Magnetic resonance imaging
OB/GYN: Obstetrics and gynecology
RCA: Rapid cycle approaches
U.S.: United States
