# Supplementary material for: Protocol for a pragmatic stepped wedge cluster randomized clinical trial testing behavioral economic implementation strategies to increase supplemental breast MRI screening among patients with extremely dense breasts
Source: Implement Sci. 2023 Nov 24;18:65. doi: 10.1186/s13012-023-01323-x (PMC10668465; doi:10.1186/s13012-023-01323-x)
Supplement: Supplementary file 1 — Additional file 1. Patient Education Materials. [file 13012_2023_1323_MOESM1_ESM.docx]

**Supplemental File 1.** Additional Questions & Answers Content for Patients

**What is breast density?**

There are three different types of breast tissue: fibrous, glandular, and fatty tissue. Breast density compares the amounts of fibrous and glandular tissue to the amount of fatty tissue. Breast density is detected by mammograms, and about half of women older than 40 have dense breasts, and about 1 in 10 women have extremely dense breasts. Breast density can hide tumors and other abnormalities in the breast, making them more difficult to find and treat.

**What is a breast MRI?**

Magnetic Resonance Imaging (MRI) is a type of scan that uses a powerful magnet and radio waves to create 3-D images. Breast MRI uses a dedicated breast coil that makes it easier to take clear and detailed images of the breast. Breast MRI can be used to diagnose and stage breast cancer and other breast conditions. It is a non-invasive imaging test, which means no tools are used that penetrate your skin.

**What happens during a breast MRI?**

For most breast MRIs, you will need to get an injection of contrast, which is a solution that helps show the condition of the breast more clearly. Your care team will place an IV in your arm before the study begins. Then, an injection of contrast agent will be given via the IV throughout the breast MRI. During a breast MRI, you will lay face down on a table that moves through a tube-shaped structure where images of the breasts are created. Breast MRI does not use radiation or X-ray exposure and is considered a non-invasive imaging test. Our expert breast imaging radiologists will then interpret the study, and they will send the results to you and your doctor.

**Why do I need a breast MRI in addition to my mammogram?**

People with dense breasts have 3 to 5 times greater risk of developing breast cancer as compared to those without dense breasts. A mammogram helps to detect cancer early, but it is not a perfect test. Dense tissue can hide breast cancers on mammograms, and recent studies have found that screening with Magnetic Resonance Imagining (MRI) in addition to regular mammography can improve detection of small, invasive cancers that may not be detected by mammography alone. This is important because detecting breast cancer early means less treatment, as well as fewer or no breast cancer-related surgeries and improved survival.

**What are the risks of MRI screening?**

Like other tests, an MRI can sometimes result in false positives, where extra tests or biopsies are performed but no cancer is found. These extra tests could lead to additional costs following the MRI. In rare cases, the dye used during MRIs can cause an allergic reaction. Additionally, MRI machines can cause some people to feel claustrophobic, which means they feel discomfort or fear in closed spaces.

**Does insurance cover breast MRI after a mammogram?**

In January 2022, Pennsylvania passed a law that requires MRI screening to be covered by insurance for those with extremely dense breasts. However, be mindful that you might be responsible for certain copay or co-insurance charges based on your specific insurance plan.

**What costs may I be responsible for when getting a breast MRI?**

It is important to note that even though full MRI screening is covered by insurance under Pennsylvania law for patients with extremely dense breasts, you might still be responsible for some costs depending on your specific insurance plan. Costs for patients who have private insurance plans can vary greatly, especially if your plan has a high deductible. It is important that you check with your insurance plan before scheduling a breast MRI. You can call Financial Clearance to discuss your specific costs at 267-414-2760 or contact the Patient Financial Advocacy team at PatientFinancialAdvocacy@uphs.upenn.edu or 215-662-3505.

**How do I schedule a breast MRI?**

To schedule your supplemental breast MRI screening, contact your primary care provider or OB/GYN and ask them to order a breast MRI screening.

This information can be found at the following website: <https://www.pennmedicine.org/for-patients-and-visitors/find-a-program-or-service/radiology/breast-imaging/faqs-breast-imaging-mammogram-mri#goto-BreastMRI>
