# Supplementary material for: Protocol for a pragmatic stepped wedge cluster randomized clinical trial testing behavioral economic implementation strategies to increase supplemental breast MRI screening among patients with extremely dense breasts
Source: Implement Sci. 2023 Nov 24;18:65. doi: 10.1186/s13012-023-01323-x (PMC10668465; doi:10.1186/s13012-023-01323-x)
Supplement: Supplementary file 2 — Additional file 2. Clinician Education Material. [file 13012_2023_1323_MOESM2_ESM.pdf]

Penn Radiology

Penn Medicine

Breast Density and Supplemental Breast Screening Options

Why Supplemental Screening?

- Women with dense breasts have 3-5-fold increased risk for breast cancer compared to women without dense breasts. [1,2]
- Dense breast tissue can "mask" small tumors, leading to reduced mammography sensitivity. [1,2]
- The rate for interval invasive cancers is 0.87 per 1000 for women with extremely dense breasts screened with digital breast tomosynthesis (DBT). [3]

Why Breast Magnetic Resonance Imaging (MRI)?

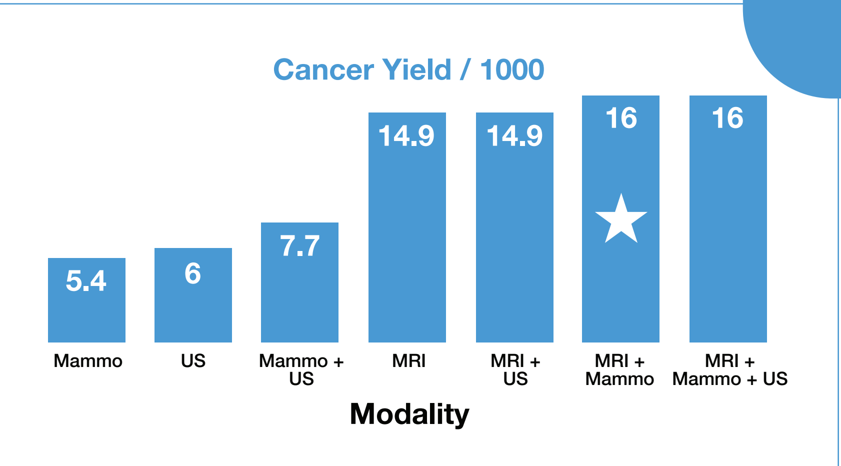

Figure 1: Cancer Yield among 678 high risk women screened with mammography, ultrasound (US), and MRI read independently and in combination

KEY POINTS

- Patients with dense breasts have increased breast cancer risk
- Mammography has poorer sensitivity for these patients
- MRI is superior to ultrasound for breast supplemental screening
- PA law mandates insurance coverage of MRI for patients with extremely dense breasts
- Order breast MRI for patients with extremely dense breasts

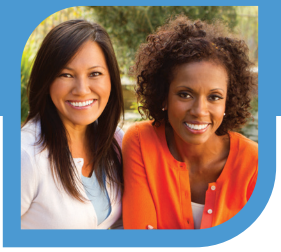

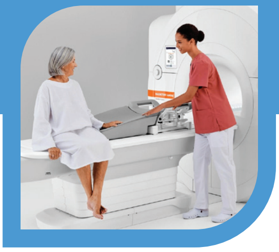

Supplemental breast MRI screening improves detection of invasive cancers that are not detected by mammography[4] and reduces the interval cancer rate by 50% among women with **extremely dense breasts.**[5]

Are There Any Contraindications to Breast MRI?

Some contraindications to breast MRI can be impaired renal function, metal bone replacements or other implants, pregnancy, allergy to gadolinium contrast agents.

What are the risks of MRI screening?

MRIs do carry risks, most notably an increased likelihood of false positives, which may prompt unnecessary biopsies and downstream costs. Other risks include the possibility of overdiagnosing cancers that might not pose a risk to individuals in their lifetime, as well as rare adverse reactions to the injection of contrast materials.

What Test Should I Order?

Consider supplemental breast MRI screening ***(MR BREAST W AND WO CONTRAST BILATERAL, CPT 77049)*** for patients with extremely dense breasts. When placing the MRI order, include the diagnosis "dense breasts" and select the indication for exam "supplemental breast cancer screening, dense breasts".

Is Supplemental Screening Covered by Insurance?[6]

Based on recent data, a law was passed in Pennsylvania mandating insurance coverage of one supplemental screening with breast MRI per year for women with extremely dense breasts. Currently, copays and coinsurance may be charged however, in the near future, Pennsylvania law will be removing out of pocket charges. We recommend for patients to contact their insurance company to know their financial responsibility.

What if My Patient Cannot Afford the Out-Of-Pocket Costs?

Contact the **Patient Financial Advocacy team** at *PatientFinancialAdvocacy@uphs.upenn.edu* or **215-662-3505**.

Patients can also call **Radiology Financial Clearance** at **267-414-2760** to get a cost estimate.

[1]PMID: 24816206 [2]PMID: 30206060 [3]PMID: 35699706 [4]PMID: 20177029. [5]PMID: 31774954 [6]https://www.legis.state.pa.us/cfdocs/legis/li/uconsCheck.cfm?yr=2020&sessInd=0&act=52.
